# Computed Tomographic Characteristics of Feline Renal Cell Carcinoma and Renal Lymphoma: A Comparative Analysis

**DOI:** 10.3390/vetsci12040360

**Published:** 2025-04-12

**Authors:** Choye Shin, Kidong Eom, Jaehwan Kim, Aryung Nam, Yewon Joo, Inseong Jung, Jihee Park, Noh-won Park, Minsu Lee, Dong-gil Lee, Seunghak Yeo, Heejung Yu

**Affiliations:** 1 Department of Veterinary Medical Imaging, College of Veterinary Medicine, Konkuk University, Seoul 05029, Republic of Korea; tlschdl000@konkuk.ac.kr (C.S.); eomkd@konkuk.ac.kr (K.E.); aryung@konkuk.ac.kr (A.N.);; 2 Royal Animal Medical Center, Seoul 02140, Republic of Korea; 3 Joy Animal Medical Center, Gyeonggi-do 15382, Republic of Korea; 4 Nowon N Animal Medical Center, Seoul 01704, Republic of Korea; 5 SD Animal Medical Center, Seoul 04580, Republic of Korea; 6 Haeden Animal Medical Center, Gyeonggi-do 11813, Republic of Korea; 7 Bien Animal Medical Center, Gyeonggi-do 14675, Republic of Korea

**Keywords:** feline, computed tomography, renal tumor

## Abstract

This retrospective, multicenter study aimed to identify key computed tomography (CT) features that differentiate renal cell carcinoma (RCC) from lymphoma, the two most common feline renal neoplasms. CT images from 25 cats with renal tumors (15 RCC and 10 lymphoma) were evaluated. Of the RCC cases, 10 were diagnosed through histopathology and 5 through FNA. Among the lymphoma cases, two were extranodal lymphoma, including one case of primary renal lymphoma, and eight were gastrointestinal (GI) lymphoma with renal involvement. Qualitative features (tumor distribution, growth pattern, and enhancement characteristics) and quantitative parameters (tumor size and attenuation values) from triphasic or single post-contrast-enhanced CT scans were analyzed and compared. RCC typically presented as unilateral (93.3%) masses with expansile growth (73.3%) and heterogeneous enhancement among contrast-enhancing tumors (100%). Lymphoma more often showed bilateral involvement (60%), infiltrative growth (50%), and homogeneous enhancement (90%) (*p* < 0.001). Tumor vessel enhancement was observed exclusively in RCC. Compared with lymphoma, RCC demonstrated significantly higher attenuation values in the late nephrographic/early excretory phase. While histopathology remains the gold standard, this study provides a detailed CT analysis of these tumors, specifically within a feline population.

## Simple Summary

Kidney tumors in cats, though relatively uncommon, represent an important clinical entity in feline medicine. Among the various types, renal cell carcinoma (RCC) and lymphoma are the two most commonly diagnosed renal neoplasms. The goal was to identify key imaging features that distinguish these tumors. The study found that RCC usually appeared as a unilateral, solitary mass with heterogeneous enhancement and visible tumor vessels. In contrast, lymphoma commonly presents with bilateral involvement, multiple lesions, and homogeneous enhancement patterns. Quantitative analysis revealed that RCC exhibited significantly higher attenuation values in the late nephrographic/early excretory phase of contrast imaging compared to lymphoma. These findings are important because they improve early and accurate diagnosis, which can lead to better treatment decisions and improved care for cats with kidney tumors.

## 1. Introduction

Primary renal tumors are rare in cats, accounting for approximately 2.5% of all feline neoplasms [1]. Among these, lymphoma is the most frequently diagnosed renal tumor in cats, followed by renal cell carcinoma (RCC). Accurate differentiation between these tumor types is crucial as they exhibit distinct biological behaviors and require different treatment approaches [2].

Diagnostic imaging is essential for evaluating kidney diseases. In veterinary medicine, ultrasonography (US) is frequently used as the initial diagnostic tool; however, computed tomography (CT) has demonstrated superior detection of both inflammatory and neoplastic nodules compared to US [3]. Additionally, CT offers distinct advantages in differentiating normal renal tissue from pathological processes by providing detailed anatomical analysis and optimized visualization of the renal parenchyma and collecting system [4,5]. On non-contrast CT, normal canine renal parenchyma typically demonstrates homogeneous soft-tissue attenuation, with clear differentiation between cortex and medulla observed after contrast administration. In contrast, renal neoplasms exhibit characteristic alterations in tissue density, enhancement patterns, and morphological features that differ significantly from normal parenchyma [6]. These differences form the basis for diagnostic interpretation and tumor characterization.

In human medicine, the roles of CT and magnetic resonance imaging (MRI) in characterizing renal masses are well established. Both modalities achieve greater than 95% accuracy and are highly effective in detecting retroperitoneal lymphadenopathy. Additionally, distant metastases to the liver and bone are generally well visualized using these techniques [7,8]. Given the diverse biological behaviors and prognoses associated with RCC subtypes (clear cell, papillary, and chromophobe), triphasic contrast-enhanced CT is a valuable tool for differentiating renal tumors and identifying their specific histological subtypes [9,10]. Unlike in humans, feline RCCs are categorized into three main subtypes according to their primary histological characteristics: papillary, tubular, and solid [11].

Despite the clinical importance of differentiating feline renal tumors, comprehensive studies utilizing advanced imaging modalities such as triphasic contrast-enhanced CT are limited in the veterinary literature. Previous studies have primarily focused on individual tumor types or have been conducted in other species such as dogs and humans. This study retrospectively evaluated the qualitative and quantitative CT features of feline RCC and lymphoma using a triphasic or a single post-contrast-enhanced CT protocol. The aim was to identify key imaging characteristics that can aid in differentiating these two common renal neoplasms in cats. Additionally, the study explored the spectrum of CT features observed in both RCC and lymphoma, including typical and atypical presentations, to provide a comprehensive understanding of their imaging appearances.

## 2. Materials and Methods

### 2.1. Study Design and Case Selection

This retrospective multicenter study was conducted using medical databases from six veterinary institutions: Konkuk University Veterinary Medical Teaching Hospital, Royal AMC, Joy AMC, SD AMC, Nowon N AMC, and Haedeun AMC. The study period spanned from April 2016 to December 2024. The inclusion criteria for case selection were as follows: (1) the presence of renal masses identified on CT scans, (2) the availability of full thoracic and abdominal contrast-enhanced CT scans, and (3) the histological or cytological confirmation of renal tumors. In the RCC group, cases were diagnosed through histopathological examination following nephrectomy (*n* = 10) or ultrasound-guided FNA (*n* = 5). All the lymphoma cases were diagnosed using ultrasound-guided FNA (*n* = 9), except for one case that was confirmed through histopathological examination (*n* = 1). All the cats included in this study had renal masses initially identified through other diagnostic modalities, such as radiographs or ultrasonography, or were detected by palpation. Cats with poor-quality CT images or inconclusive histopathological or cytological results were excluded from the analysis. The collected data included signalment (breed, age, sex, and body weight), clinical findings from physical examinations, hematological results, and clinical signs. All the cats underwent abdominal contrast-enhanced CT for a comprehensive evaluation of the renal masses.

### 2.2. Computed Tomography Scan Techniques

CT examinations were performed using multiple scanners, including the Aquilion 64 and Activion 16 (Canon Medical Systems Corporation, Tochigi, Japan), Emotion 16 (Siemens Healthineers, Erlangen, Germany), Revolution ACT and Optima CT660 (GE Healthcare, Chicago, IL, USA), and Aquilion Lightning 160 (Canon Medical Systems Corporation, Tochigi, Japan). The scanners had detector rows ranging from 16 to 160. The CT image acquisition parameters included slice thickness (0.75–2.5 mm), helical pitch factors (0.8–1.35), rotation times (0.5–1.0 s), kVp values (120–130), and mAs values (60–170). For contrast-enhanced studies, a nonionic contrast medium (iohexol 350 mg iodine/mL; Omnipaque, GE Healthcare) was administered either manually or via a power injector at a rate of 2–2.5 mL/s through the cephalic vein, with a dose of 2.5 mL/kg. For cases that utilized a three-phase protocol, the bolus-tracking method was employed. Corticomedullary, nephrographic, and delayed nephrographic/early excretory phases were acquired with delay times of 5, 40, and 70 s, respectively. In cases where a three-phase contrast-enhanced protocol was not performed, post-contrast images were acquired 60–80 s after contrast injection and classified as the delayed nephrographic/early excretory phase [6,12,13]. The late nephrographic/early excretory phase was categorized based on the presence of contrast medium within the renal pelvis and, in some cases, progression into the ureters. Among the 25 cases, 12 cats (6 RCC and 6 lymphoma) underwent triphasic contrast-enhanced CT. Of the remaining cases, 13 (9 RCC and 4 lymphoma) had pre- and delayed nephrographic/early excretory phase CT studies. Two RCC cases were excluded from the analysis of enhancement patterns and vascular-related evaluations (tumor vessel enhancement and vascular invasion) due to the lack of internal contrast enhancement.

### 2.3. Image Analysis

An image analysis was conducted based on previously published studies on canine renal tumors using CT [6,14]. This study evaluated various qualitative and quantitative CT parameters to characterize renal tumors. Renal tumor involvement was categorized as either unilateral or bilateral based on the affected kidney(s). The tumors were further categorized as solitary or multiple depending on the number of distinct lesions identified in one or both kidneys. The growth pattern of renal tumors was categorized into three types: expansile, infiltrative, and combined. Expansile growth was defined as a tumor that bulges outward, distorting the normal renal contour. In contrast, infiltrative growth was characterized by poorly defined tumor margins that may enlarge the kidney but usually maintain the reniform shape [8]. A combined growth pattern exhibited both expansile and infiltrative features simultaneously. The spatial enhancement patterns of the renal mass were subjectively classified as homogeneous (uniform appearance) or heterogeneous (varied attenuation) [14]. The CT attenuation values (AVs) were analyzed from the corticomedullary to the late nephrographic/early excretory phases to assess the time-course contrast enhancement pattern. The enhancement patterns were categorized as either plateau or progressive. A plateau pattern was characterized by a gradual increase in the AV of the renal masses up to the nephrographic phase, followed by a decrease in the late nephrographic/early excretory phase. In contrast, a progressive pattern exhibited a steady increase in AVs throughout all three phases of the CT study [14,15]. Tumor vascularity was assessed based on the presence of vascular enhancement. Lesions were considered hemorrhagic if they appeared as hyperattenuating areas on non-contrast CT scans, with a CT value ranging from 40 to 70 Hounsfield Units (HU) [16,17]. Necrosis was identified based on the presence of ill-defined, hypoattenuating areas that lacked enhancement during the nephrographic and delayed nephrographic/early excretory phases of contrast-enhanced CT [18]. Lymphadenopathy was defined as lymph node enlargement based on prior research findings [19]. Vascular invasion into the renal vein or caudal vena cava (CVC) and intratumoral mineralization were recorded. Additionally, lung metastases were evaluated using thoracic CT images analyzed in a detailed lung window setting.

The quantitative analysis involved measuring tumor dimensions on CT images in three planes. The measurements were then assigned to length, width, and height based on their relative lengths. Renal tumor AV (in HU) was measured across the pre- and post-contrast phases. These measurements were performed three times per case by a single observer, and mean AV was calculated for each tumor type. The attenuation values on post-contrast CT studies can be affected by numerous factors including patient-related factors (body size and cardiac output) and injection-related factors (volume, concentration, and injection rate of contrast medium). To account for these variables, aorta-based corrected AV and relative values were calculated using a coefficient factor. This coefficient factor was defined as the ratio of the mean aortic AV across all the patients to the individual patient’s aortic AV, measured at the level of the renal artery branching from the aorta in each phase. Furthermore, the relative contrast enhancement values were calculated by dividing the corrected AV by the tumor AV on unenhanced CT images [20]. All the measurements were obtained by a single radiologist under the supervision of a senior radiologist with over 10 years of experience in radiology.

### 2.4. Statistical Analysis

Statistical analyses were performed to evaluate the qualitative and quantitative CT parameters of renal tumors, comparing RCC and lymphoma. Continuous variables such as tumor size and AVs (HU) were analyzed for normality using the Shapiro–Wilk test. Normally distributed data were compared using independent *t*-tests, whereas non-normally distributed data, including aorta-based corrected and relative contrast enhancement AV, were analyzed using the Mann–Whitney U test. Categorical variables, such as renal tumor involvement, number of lesions, spatial and time-course enhancement patterns, presence of vascular invasion, intratumoral mineralization, lymphadenopathy, and pulmonary metastases, were compared using Fisher’s exact test. Statistical significance was set at a *p*-value < 0.05.

## 3. Results

### 3.1. Study Population Characteristics

A total of 25 cats with renal tumors were included in this study. The study population comprised 15 cats (60%) with RCC and 10 cats (40%) with renal lymphoma. Among the lymphoma cases, two were classified as extranodal lymphoma, with one involving the nasal cavity and brain and the other diagnosed as primary renal lymphoma. The remaining eight cases were diagnosed with GI lymphoma with variable extraintestinal involvement including kidney(s).

The study population consisted of domestic shorthaired (*n* = 21), Turkish Angora (*n* = 2), Bengals (*n* = 1), and Ragdoll cats (*n* = 1). The sex distributions were as follows: castrated males (*n* = 12, 48%), spayed females (*n* = 10, 40%), and intact females (*n* = 3, 12%). The mean age of the cats with RCC (10.3 ± 2.4 years) was higher than that of those with lymphoma (8.2 ± 3.2 years), although this difference was not statistically significant (*p* = 0.069). The mean body weight was similar between the RCC (4.2 ± 1.5 kg) and lymphoma (4.2 ± 1.2 kg) groups (*p* = 0.991).

The most common clinical symptoms were anorexia (RCC, *n* = 9; lymphoma, *n* = 7) and lethargy (RCC, *n* = 5; lymphoma, *n* = 3). Weight loss was observed in the RCC (*n* = 4) and lymphoma cases (*n* = 4). Urinary signs (dysuria and foamy urine) were rare, with only one case reported in each group (*n* = 1). GI symptoms (vomiting, diarrhea, hematemesis, and hematochezia) were significantly more prevalent in the lymphoma cases (*n* = 7) than in the RCC cases (*n* = 3) (*p* = 0.034).

The laboratory results revealed the following findings: Azotemia was prevalent in both the RCC (7/13, 53.8%) and lymphoma (5/9, 55.6%) cases. Polycythemia was significantly more common in the RCC (8/14, 57.1%) than in the lymphoma cases (0/9, 0%) (*p* = 0.007). Conversely, anemia was more frequent in the lymphoma (4/9, 44.4%) than in the RCC cases (1/13, 7.7%). Calcium abnormalities were rare in both groups, with hypercalcemia observed in the lymphoma (2/8, 25%) and RCC (1/13, 7.7%) cases. Urinalysis findings, though based on a limited number of cases, showed hematuria in the patients with lymphoma (4/5, 80%) and RCC (2/6, 33.3%). Proteinuria was observed in the lymphoma (3/5, 60%) and RCC (2/6, 33.3%) samples.

### 3.2. Qualitative CT Features

**Figure 1:**
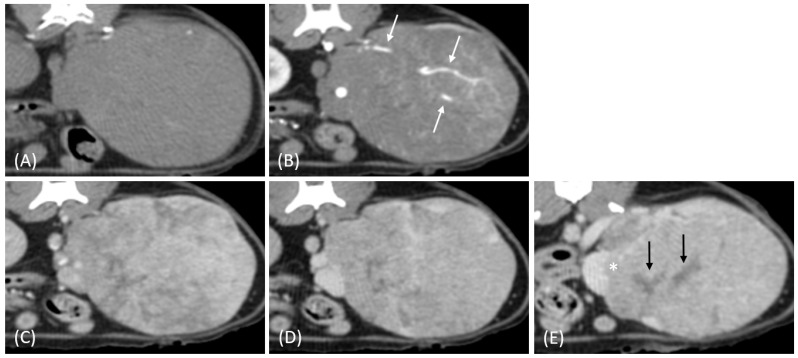
(**A**) Pre-contrast, (**B**) corticomedullary phase, (**C**) nephrographic phase, and (**D**,**E**) late nephrographic/early excretory phase contrast-enhanced computed tomography (CT) images of a cat with a unilateral left renal cell carcinoma. The mass is heterogeneous, with tumor vessel enhancement (white arrow) noted in the corticomedullary phase. Progressive enhancement is observed, along with internal necrosis (black arrow). The renal vein is enlarged with tumor invasion (asterisk), but no invasion into the caudal vena cava (CVC) is seen. The tumor encases the renal artery, though definitive arterial invasion is not apparent.

**Figure 2:**
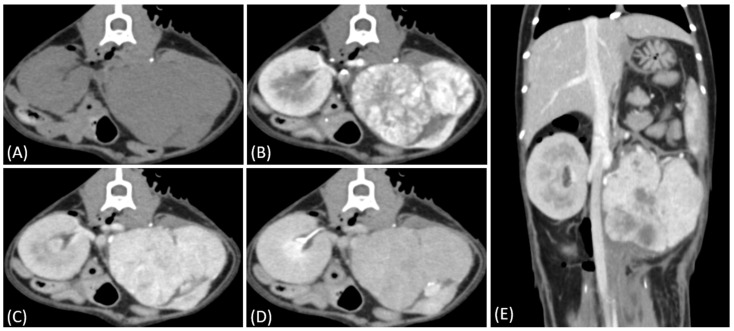
(**A**) Pre-contrast, (**B**) corticomedullary phase, (**C**) nephrographic phase, and (**D**,**E**) late nephrographic/early excretory phase contrast-enhanced computed tomography (CT) images of a cat with a unilateral left renal cell carcinoma. The tumor demonstrates predominantly heterogeneous and plateau enhancement patterns throughout the phases, with focal areas of intense enhancement visible during the corticomedullary phase.

**Figure 3:**
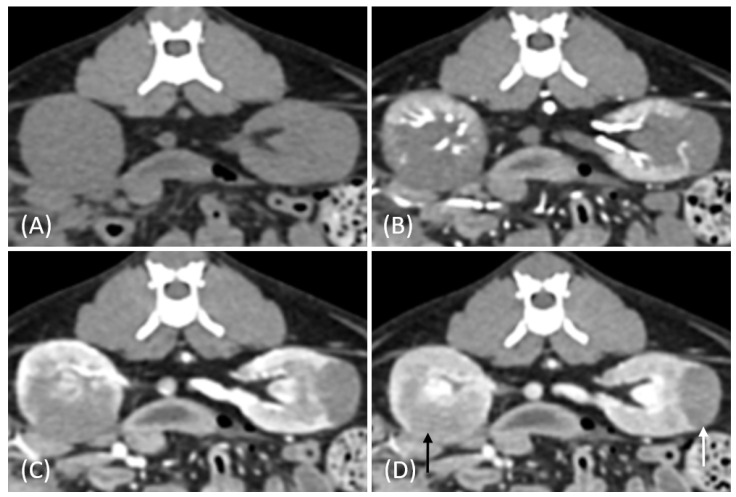
(**A**) Pre-contrast, (**B**) corticomedullary phase, (**C**) nephrographic phase, and (**D**) delayed nephrographic/early excretory phase contrast-enhanced computed tomography (CT) images of a cat with bilateral renal and small intestinal lymphoma. The right kidney shows an infiltrative mass (black arrow), while the left kidney has an expansile mass (white arrow). Both tumors are homogeneous and hypodense, demonstrating progressive enhancement.

**Figure 4:**
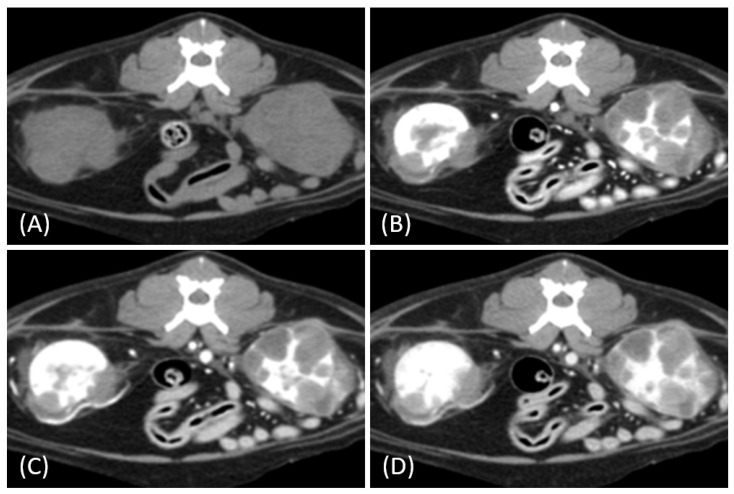
(**A**) Pre-contrast, (**B**) corticomedullary phase, (**C**) nephrographic phase, and (**D**) delayed nephrographic/early excretory phase contrast-enhanced computed tomography (CT) images of a renal cell carcinoma. The tumors are bilateral, predominantly demonstrating an expansile growth pattern. They appear relatively homogeneous and hypoattenuating, resembling the imaging characteristics of multiple lymphoma masses. Progressive enhancement is observed throughout the phases. These findings highlight the overlapping imaging features between renal cell carcinoma (RCC) and lymphoma in certain cases.

**Figure 5:**
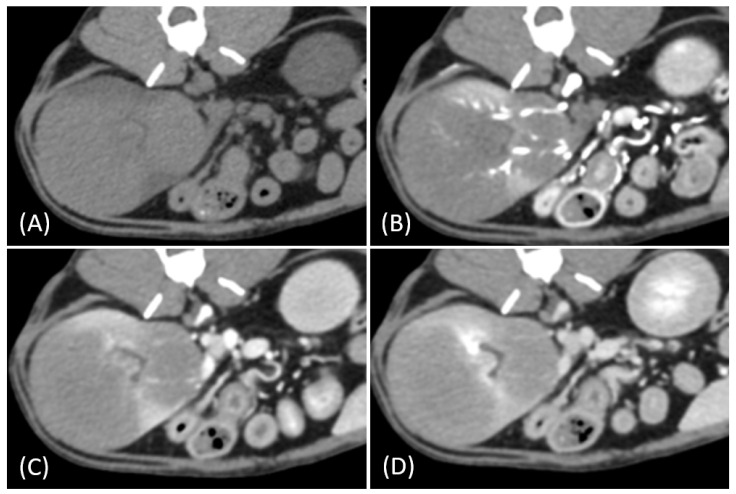
(**A**) Pre-contrast, (**B**) corticomedullary phase, (**C**) nephrographic phase, and (**D**) delayed nephrographic/early excretory phase contrast-enhanced computed tomography (CT) images of a cat with primary renal lymphoma. The right kidney shows a unilateral, infiltrative mass with noticeable hypertrophy. The tumor is homogeneous and hypodense, displaying progressive enhancement.

Regarding tumor distribution, RCC predominantly presented as unilateral lesions (14/15, 93.3%) (Figure 1 and Figure 2) with left kidney involvement being more common (10/15, 66.7%) than right kidney involvement (4/15, 26.7%). In contrast, lymphoma exhibited a significant tendency for bilateral involvement (6/10, 60%) (Figure 3) (*p* = 0.007). Additionally, one case of bilateral RCC (Figure 4) and one of unilateral primary renal lymphoma (Figure 5) were identified. The number of lesions also differed significantly between RCC and lymphoma (*p* < 0.001). RCC typically presented as a solitary mass (14/15, 93.3%), whereas lymphoma more commonly exhibited multiple lesions (8/10, 80%).

Tumor growth patterns differed significantly between RCC and lymphoma cases (*p* = 0.015). RCC predominantly exhibited an expansile growth pattern (11/15, 73.3%) (Figure 1 and Figure 2), while infiltrative patterns were observed in three cases (3/15, 20%) and a combined pattern in one case (1/15, 6.7%). In contrast, lymphomas showed more variable patterns, with infiltrative growth being the most common (5/10, 50%), followed by combined (3/10, 30%) and expansile patterns (2/10, 20%) (Figure 3).

**Figure 6:**
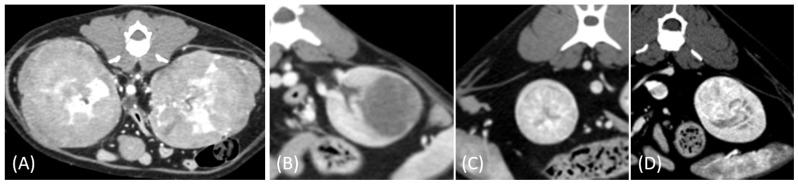
Contrast-enhanced computed tomography (CT) images of renal tumors in different cats. (**A**) Lymphoma with a heterogeneous and irregular appearance. (**B**) Papillary renal cell carcinoma (RCC) presents as a relatively hypoenhancing mass, mimicking the solitary hypoenhancing mass observed in the lymphoma. Partially nonenhancing hypoattenuating regions corresponded to extensive internal necrosis observed in the histopathological analysis. (**C**,**D**) Incidental findings of small RCCs in two different cats demonstrating increased vascularity and heterogeneity without significant alteration of kidney shape.

The spatial enhancement pattern differed significantly between the two tumor types (*p* < 0.001), with RCC typically showing heterogeneous enhancement (13/13, 100%) and lymphomas displaying homogeneous enhancement (9/10, 90%). However, one lymphoma case demonstrated a heterogeneous enhancement pattern (Figure 6A). Tumor vessel enhancement in the corticomedullary phase was observed exclusively in the RCC cases (4/5, 80%) (Figure 1B), while none of the lymphoma cases showed this feature (0/6, 0%) (*p* = 0.015).

The time-course enhancement pattern analysis revealed different characteristics between the RCC and lymphoma cases. Among the RCC cases with available three-phase enhancement data (*n* = 5), three cases (3/5, 60%) showed a progressive enhancement pattern (Figure 1), while two cases (2/5, 40%) demonstrated a plateau pattern (Figure 2). By contrast, all the lymphoma cases with available data (*n* = 6) exhibited progressive enhancement patterns. However, the difference in the time-course enhancement patterns between the two tumor types was not statistically significant (*p* = 0.182).

The internal characteristics showed marked differences between the two tumor types, with necrosis being significantly more prevalent in RCC (14/15, 93.3%) (Figure 1E) than in lymphoma (1/10, 10%) (*p* < 0.001). Hemorrhage was also more commonly observed in RCC (8/15, 53.3%) compared to lymphoma (1/10, 10%) (*p* = 0.040). In some RCC cases (*n* = 5), gross and histopathological analysis confirmed internal necrosis and hemorrhage, which corresponded to the hypoattenuating regions observed on the CT images (Figure 6B).

Additional findings included regional abdominal lymphadenopathy, which was more common in lymphoma (5/10, 50%) than in those with RCC (1/15, 6.7%) (*p* = 0.023). Vascular invasion (1/13, 7.7%) (Figure 1E) and intratumoral mineralization (4/15, 26.7%) were observed only in the RCC cases. A comprehensive comparison of the qualitative CT features of feline RCC and lymphoma is presented in Table 1.

### 3.3. Quantitative CT Features

The quantitative analysis revealed significant differences in tumor sizes and AVs between the RCC and lymphoma cases. The RCC mean tumor dimensions were significantly larger than lymphomas, with length (4.9 ± 2.0 vs. 2.7 ± 1.2 cm, *p* = 0.006), width (4.3 ± 1.5 vs. 2.4 ± 1.0 cm, *p* = 0.002), and height (3.7 ± 1.3 vs. 2.0 ± 1.9 cm, *p* = 0.001).

Pre-contrast AVs were similar between RCC (42.5 ± 6.2 HU) and lymphoma (42.3 ± 5.7 HU, *p* = 0.942). However, after contrast administration, RCC demonstrated higher AV than lymphoma, with a statistically significant difference observed only in the delayed nephrographic/early excretory phase (175.2 ± 72.8 vs. 89.9 ± 24.2 HU, *p* = 0.001). For detailed AVs across all the phases, refer to Table 2. Individual RCC cases in our study showed maximum peaks of 239.4 HU in the corticomedullary phase, 276.3 HU in the nephrographic phase, and 298.4 HU in the delayed nephrographic/early excretory phase. Among the RCC cases with available histopathological data (*n* = 10), papillary RCC was diagnosed in two, tubulopapillary RCC in one, and tubular RCC in seven. When categorized as papillary and non-papillary subtypes, the mean AV on pre- and delayed nephrographic/early excretory phase CT were 36.9 ± 8.4 HU and 101.9 ± 31.9 HU for papillary RCC, and 44.6 ± 4.7 HU and 228.9 ± 81.4 HU for non-papillary RCC, respectively. The two papillary RCC cases had individual delayed nephrographic/early excretory phase HU values of 124.5 and 79.3, which were the lowest among all the RCC cases in this study. Interestingly, the tubulopapillary RCC case exhibited an intermediate value (the third lowest among all RCC cases). The HU values for papillary RCC showed similarities to those of lymphoma.

**Figure 7:**
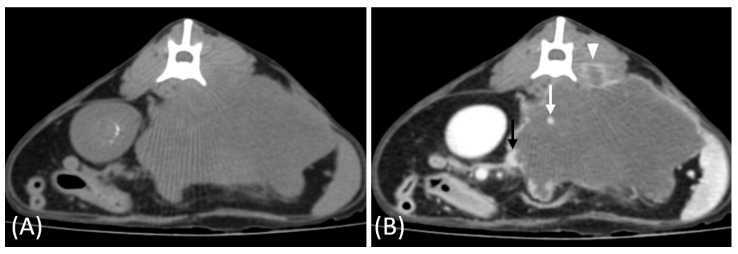
(**A**) Pre-contrast and (**B**) late nephrographic/early excretory phase contrast-enhanced computed tomography (CT) images of a renal cell carcinoma (RCC). The image shows a large renal mass with rim enhancement and no internal enhancement. Vascular structures such as the aorta (white arrow) and CVC (black arrow) are visible; however, the renal artery and vein are not clearly identifiable. Tumor invasion into adjacent musculature is observed (arrowhead).

In the two cats that were excluded, the RCC tumors showed a distinct imaging pattern with rim enhancement but no internal enhancement on contrast-enhanced CT (Figure 7). The attenuation values of the tumors were approximately 23 HU and 33 HU across both the pre- and post-contrast phases.

**Figure 8:**
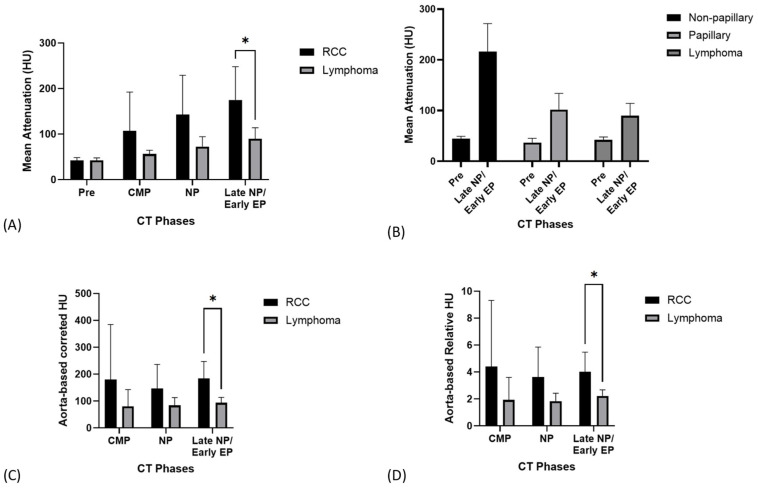
Computed tomography (CT) attenuation values of renal cell carcinoma (RCC) and lymphoma across different phases: (**A**) Mean attenuation values (Hounsfield Units, HU) of RCC and lymphoma. A significant difference was observed in the delayed nephrographic/early excretory phase (*p* < 0.05). (**B**) Mean attenuation values of RCC subtypes (non-papillary, papillary) and lymphoma in the pre- and delayed nephrographic/early excretory phases. (**C**) Aorta-based corrected HU values for RCC and lymphoma, with a significant difference in the delayed nephrographic/early excretory phase (*p* < 0.05). (**D**) Aorta-based relative HU values for RCC and lymphoma. A significant difference was noted in the delayed nephrographic/early excretory phase (*p* < 0.05). HU = Hounsfield Unit; RCC = renal cell carcinoma; CT, computed tomography; CMP = corticomedullary phase; NP = nephrographic phase; EP = excretory phase. * statistically significant result.

In this study, the mean aortic AV for all the patients was 901.1 HU, 275.8 HU, and 226.9 HU for the corticomedullary, nephrographic, and delayed nephrographic/early excretory phases, respectively. An analysis of aorta-based corrected AV showed significantly higher values in RCC than in lymphoma during the delayed nephrographic/early excretory phase (184.1 ± 63.2 vs. 93.7 ± 20.4 HU, *p* < 0.001). Similarly, aorta-based relative contrast enhancement values were also elevated in RCC (4.3 ± 1.2 vs. 2.2 ± 0.5, *p* < 0.001). The pre- and post-contrast CT AV of the renal tumors is shown in Figure 8 and Table 2.

## 4. Discussion

This study identifies different imaging characteristics that differentiate feline RCC from lymphoma. Previous studies have explored the CT characteristics of primary renal tumors in dogs and cats, providing valuable insights into their imaging features. One study compared the CT findings of RCC, lymphoma, and hemangiosarcoma in dogs, while another study examined the CT features of RCC, lymphoma, and nephroblastoma in both dogs and cats [6,14]. However, the latter study included only lymphoma cases for feline renal tumors. Therefore, a comprehensive comparison of the two most common feline renal tumor types was conducted.

In our study, renal tumor involvement showed distinct patterns in RCC and lymphoma. RCC predominantly presented as solitary and unilateral lesions, with a predilection for the left kidney, whereas lymphoma showed a higher tendency for multiple and bilateral involvement, although unilateral cases were also observed. These findings align with recent studies on feline renal tumors [21,22], suggesting that unilateral involvement in renal lymphoma may be more common than previously recognized.

In our study, one feline presented with primary renal lymphoma, which is extremely rare in cats, with a reported incidence of ˂ 1% of all feline renal neoplasms and 3.6% of all feline lymphoma cases [23,24]. Interestingly, this case showed unilateral involvement differing from the typical bilateral presentation of feline renal lymphoma [14,25]. While primary renal lymphoma is well documented in humans, there is limited literature on its occurrence in cats, with most cases presenting with renal involvement secondary to systemic lymphoma [24,26]. This case highlights the importance of considering primary renal lymphoma in the differential diagnosis of unilateral renal masses, even in the absence of systemic involvement.

In contrast, one RCC case in our study presented with multiple bilateral renal masses displaying relatively low contrast enhancement, posing a diagnostic challenge in differentiating it from renal lymphoma. This finding is consistent with those of previous studies, which indicate that multiple synchronous RCCs, particularly papillary and chromophobe subtypes, can mimic multifocal renal lymphoma [27]. The diagnostic complexity arises because papillary RCC typically appears as a homogeneous, hypovascular mass with progressive contrast enhancement, especially in small-sized tumors (≤3 cm) [28]. In cats, our results further support this notion, revealing similarly low HU values between papillary RCC and lymphoma in the delayed nephrographic/early excretory phase. These findings underscore the importance of histopathological confirmation for a definitive diagnosis, particularly in cases with atypical presentation.

RCC predominantly exhibits an expansile growth pattern, whereas lymphoma shows variable growth patterns, with infiltrative growth being the most common. This difference in growth patterns reflects the distinct pathophysiology of these tumors. RCC expands and displaces normal renal tissue, whereas lymphoma grows through the diffuse infiltration of malignant lymphocytes into the renal interstitium, leading to nephromegaly without distorting the normal renal anatomy [29]. While RCC typically demonstrates a radial expansion with focal bulging of the renal contour and pseudo-capsule formation [30], our findings suggest that growth patterns can vary depending on tumor size and stage at detection. RCCs can grow to a large size before becoming clinically evident due to their retroperitoneal location and the rich vascular supply of the kidneys [31]. This aligns with our results that the renal masses in the RCC cases were significantly larger than those in the lymphoma cases across all the dimensions (*p* < 0.05). Notably, two cases of incidentally detected small RCCs in this study exhibited an infiltrative-like pattern while preserving the reniform contour. These findings suggest that early-stage RCCs can present with subtle growth patterns that preserve the renal contour and remain clinically silent.

RCCs predominantly demonstrate heterogeneous enhancement, which can be attributed to necrosis and hemorrhage, as confirmed by gross and histopathological examinations in some cases. The histologically confirmed necrotic regions corresponded to the hypoattenuating areas observed on CT. This finding aligns with previous reports associating the heterogeneous appearance of RCC with tumor necrosis and intratumoral hemorrhage [6,30], reflecting the rapid growth and variable vascularity of RCC [8,32]. However, not all the cases had histological confirmation due to sampling limitations or the absence of these features in the examined sections. Additionally, mineralization in RCC may result from necrosis and hemorrhage, which creates conditions for mineral deposition [33]. In contrast, lymphoma cases predominantly exhibit homogeneous enhancement, with significantly lower rates of necrosis and hemorrhage. The preserved renal architecture in lymphoma allows for a more uniform contrast distribution throughout the tumor [6]. The enhancement pattern shows remarkable similarities to those reported in canine renal tumors where RCC predominantly demonstrates heterogeneous enhancement (7/9, 78%), while renal lymphoma consistently exhibits homogeneous enhancement (4/4, 100%) [6]. However, one lymphoma case in our study demonstrated atypical heterogeneous enhancement likely due to the degree of renal infiltration by malignant lymphocytes [14]. This finding is consistent with human studies in which atypical CT findings, including heterogeneous attenuation, can occur in renal lymphoma with spontaneous hemorrhage or necrosis [34].

The time-course enhancement patterns of the RCC and lymphoma cases suggested potential differences. In the RCC cases with available three-phase data, all the cats showed increasing enhancement from the pre-contrast to the nephrographic phase. Three cases exhibited progressive enhancement, while two showed plateau patterns. This enhancement pattern is consistent with previous contrast-enhanced CT studies in dogs with RCC, in which a similar progressive enhancement through the nephrographic phase was observed [14]. In contrast, all the lymphoma cases with available three-phase data demonstrated a progressive enhancement pattern characterized by a gradual increase in AV across all the post-contrast phases. This homogeneous, progressive enhancement pattern in lymphomas likely reflects the uniform infiltration of malignant lymphocytes throughout the renal parenchyma.

Two RCC cases in this study exhibited a distinct imaging pattern characterized by rim enhancement without internal enhancement. This imaging feature likely reflects central necrosis or hypovascularity resulting from rapid tumor growth outpacing its blood supply. Additionally, one of the two cases demonstrated invasion into adjacent musculature, further supporting the aggressive nature of these tumors. While necrosis is a common cause of non-enhancement in tumors, other factors such as cystic changes, fibrosis, or hemorrhage may also contribute to this imaging feature [35]. Both cats succumbed to their disease within one month of diagnosis, suggesting the poor prognosis associated with this imaging pattern. Although histopathological confirmation was unavailable, these findings suggest the diagnostic value of imaging in characterizing aggressive tumors.

Tumor vessel enhancement during the corticomedullary phase was exclusively observed in the RCC cases, while the lymphoma cases lacked this feature, consistent with the findings from canine studies [6]. This distinction aligns with previous studies showing that renal lymphomas usually contain few blood vessels as they grow by separating, compressing, but preserving the remaining renal parenchymal structures rather than developing an independent vasculature [36]. The presence of tumor vessel enhancement in RCC likely reflects the hypervascular nature of these tumors, driven by neovascularization. This finding provides an important diagnostic feature for differentiating RCC from lymphomas.

Vascular invasion was detected in a relatively small proportion of the feline RCC cases, with only one case showing suspected renal vein invasion and no clear evidence of CVC involvement. This incidence is lower than that reported in both humans and dogs. In humans, vena cava infiltration occurs in 4–10% of RCC cases [37], whereas in dogs, vascular invasion has been reported in 14.3% of RCC cases, primarily involving the CVC [14]. In human RCC, tumor extension into the renal vein or CVC has significant implications for surgical planning, although it does not significantly affect the prognosis after successful nephrectomy [38]. The relatively low incidence of vascular invasion in our feline cases might reflect differences in the extent and frequency of vascular involvement compared with other species.

The high prevalence of lymphadenopathy in lymphoma compared to RCC should be interpreted with caution, as most lymphoma cases in our study represented multicentric renal involvement rather than primary renal lymphoma. Only one case was confirmed as primary renal lymphoma, suggesting that the high rate of lymphadenopathy may be influenced by the multicentric nature of systemic lymphoma. In the RCC cases, regional lymphadenopathy was relatively uncommon, with only one case (6.7%), a finding consistent with human studies where lymph node metastasis is reported in 4–10% of RCC cases [39].

Pulmonary metastasis was observed in only one RCC case (6.7%) in our study, a rate lower than previously reported rates in dogs (17.6–57.1%) [6,14,40,41]. Suspected peritoneal involvement, characterized by ascites accompanied by multiple peritoneal nodules, was observed in three cases (two RCCs and one lymphoma). While imaging features such as ascites, omental/mesenteric infiltration, and scattered nodules suggest peritoneal carcinomatosis or lymphomatosis, definitive diagnosis requires histopathological confirmation, which was not performed in this study. Prior reports have documented peritoneal carcinomatosis in feline RCC, particularly in sarcomatoid variants, although the overall incidence of peritoneal metastasis in feline RCC remains poorly documented [42]. In humans, peritoneal carcinomatosis represents an advanced stage of the disease and is associated with poor prognosis [43]. One case in our study exhibited suspected adrenal metastasis, a pattern consistent with human studies, where adrenal gland involvement occurs in approximately 4% of RCC cases [6].

The AV observed in our study showed distinct contrast enhancement patterns between feline RCC and lymphoma. RCC exhibited higher AV across the post-contrast phases, especially in the delayed nephrographic/early excretory phase, reflecting its hypervascular nature. Conversely, a less vascularized lymphoma structure resulted in consistently lower AV across all the phases. The notably higher AV in our feline cases compared with those in previous canine studies may reflect differences in predominant RCC subtypes between species. Papillary RCC is the most common subtype in dogs [41], while tubular and tubulopapillary carcinomas are the most common forms of feline RCC [11]. When analyzed by subtype, papillary RCCs appeared to have lower AV than non-papillary RCCs. This pattern aligns with human studies in which papillary RCC, known as a hypovascular subtype, typically exhibits hypoenhancement during the corticomedullary phase and peaks during the nephrographic phase. However, due to the small sample size, these observations should be considered preliminary and exploratory. Further triphasic CT studies with larger case numbers are needed to better characterize contrast enhancement patterns and facilitate the differentiation of RCC subtypes.

Although our study found differences between RCC and lymphoma in both absolute and corrected AV in the delayed nephrographic/early excretory phase that reached statistical significance in our limited sample, the importance of multiphase CT imaging in renal mass evaluation cannot be understated. The corticomedullary phase assesses tumor vascularity, the nephrographic phase offers optimal lesion characterization, and the excretory phase evaluates the collecting system involvement [20]. Therefore, complete triphasic CT imaging is essential for comprehensive lesion characterization and optimal diagnostic accuracy.

This study had some limitations. First, the relatively small number of cases with complete three-phase CT data, particularly for different RCC subtypes, limited our ability to establish definitive enhancement patterns for each tumor type. The statistical comparisons presented should be interpreted as exploratory rather than definitive. Future studies with larger sample sizes are needed to validate these preliminary patterns and potentially identify additional distinguishing features between feline renal tumor types. Second, the study was limited to RCC and lymphoma cases. Third, not all the cats underwent histopathological confirmation, and suspected metastatic lesions were not histologically verified. Finally, due to the retrospective nature of the study, optimal CT scan timing for the corticomedullary, nephrographic, and excretory phases was not consistently achieved.

## 5. Conclusions

This study demonstrates the utility of contrast-enhanced CT in characterizing feline renal tumors, particularly in differentiating between RCC and lymphoma. Distinct enhancement patterns, along with other CT features such as tumor distribution, tumor vessel enhancement, and growth patterns, provide valuable insights into renal tumors in cats. While histopathological confirmation remains crucial for definitive diagnosis, a comprehensive understanding of both typical and atypical imaging features on CT scans can significantly enhance the non-invasive assessment of feline kidney tumors.

## Figures and Tables

**Table 1:** Computed tomography (CT) features of renal cell carcinoma (RCC) and lymphoma.

CT Features	Distribution of Tumor Types		*p*-Value
	RCC	Lymphoma	
Renal tumor involvement	Right (4/15) Left (10/15), Bilateral (1/15)	Right (2/10), Left (2/10), Bilateral (6/10)	0.009 *
Number of lesions	Solitary (14/15) Multiple (1/15)	Solitary (2/10) Multiple (8/10)	<0.001 *
Growth pattern	Expansile (11/15) Infiltrative (3/15) Combined (1/15)	Expansile (2/10) Infiltrative (5/10) Combined (3/10)	0.015 *
Hemorrhage	Present (8/15)	Present (1/10)	0.040 *
Necrosis	Present (14/15)	Present (1/10)	<0.001 *
Spatial enhancement pattern	Homogeneous (0/13) Heterogeneous (13/13)	Homogeneous (9/10) Heterogeneous (1/10)	<0.001 *
Time-course enhancement pattern	Progressive (3/5) Plateau (2/5)	Progressive (6/6) Plateau (0/6)	0.182
Tumor vessel enhancement	Present (corticomedullary) (4/5)	Absent (0/6)	0.015 *
Vascular invasion	Present (1/13)	Absent (0/10)	1.000
Mineralization	Present (4/15)	Absent (0/10)	0.125
Lymphadenopathy	Present (1/15)	Present (5/10)	0.023 *
Pulmonary metastases	Present (1/15)	Present (1/10)	1.000

* statistically significant results.

**Table 2:** Pre- and post-contrast computed tomography (CT) attenuation values in renal cell carcinoma (RCC) and lymphoma.

	CT Phases	Mean Attenuation Value (HU)		*p*-Value
		RCC	Lymphoma	
	Pre-contrast	42.5 ± 6.2 (*n* = 13)	42.3 ± 5.7 (*n* = 10)	0.942
	CMP	107.0 ± 85.5 (*n* = 5)	56.2 ± 8.3 (*n* = 6)	0.255
	NP	143.2 ± 86.2 (*n* = 5)	72.7 ± 22.0 (*n* = 6)	0.142
	Delayed NP/ Early EP	175.2 ± 72.8 (*n* = 13)	89.9 ± 24.2 (*n* = 10)	0.001 *
Aorta-based corrected AV	CMP	180.2 ± 204.9 (*n* = 5)	62.9 ± 46.1 (*n* = 6)	0.126
	NP	147.0 ± 89.4 (*n* = 5)	84.1 ± 28.8 (*n* = 6)	0.195
	Delayed NP/ Early EP	184.1 ± 63.2 (*n* = 13)	93.7 ± 20.4 (*n* = 10)	<0.001 *
Aorta-based relative AV	CMP	4.1 ± 4.9 (*n* = 5)	1.4 ± 1.1 (*n* = 6)	0.126
	NP	3.6 ± 2.2 (*n* = 5)	1.8 ± 0.6 (*n* = 6)	0.150
	Delayed NP/ Early EP	4.3 ± 1.2 (*n* = 13)	2.2 ± 0.5 (*n* = 10)	<0.001 *

Values are median ± SD. * statistically significant result. CT = computed tomography; RCC = renal cell carcinoma; AV = attenuation values; CMP = corticomedullary phase; NP = nephrographic phase; EP = excretory phase.

## Data Availability

The data presented in this study are available upon request from the corresponding authors.
